# Effectiveness of Direct Pulp Capping Bioactive Materials in Dentin Regeneration: A Systematic Review

**DOI:** 10.3390/ma14226811

**Published:** 2021-11-11

**Authors:** Ermin Nie, Jiali Yu, Rui Jiang, Xiangzhen Liu, Xiang Li, Rafiqul Islam, Mohammad Khursheed Alam

**Affiliations:** 1 First Affiliated Hospital, Sun Yat-Sen University, 58 Zhong Shan Road 2nd, Guangzhou 510080, China; nieermin@mail.sysu.edu.cn (E.N.); jiangr9@mail.sysu.edu.cn (R.J.); hpyylx@163.com (X.L.); 2 Sixth Affiliated Hospital, Sun Yat-Sen University, No. 26 Yuancun Erheng Road, Guangzhou 510655, China; lxzh3@mail2.sysu.edu.cn; 3 Department of Restorative Dentistry, Faculty of Dental Medicine, Hokkaido University, Kita 13, Nishi 7, Kita-ku, Sapporo 060-8586, Japan; rony12cdc@den.hokudai.ac.jp; 4 Preventive Dentistry Department, College of Dentistry, Jouf University, Sakaka 72345, Saudi Arabia; mkalam@ju.edu.sa or; 5 Department of Dental Research Cell, Saveetha Dental College and Hospitals, Saveetha Institute of Medical and Technical Sciences, Chennai 600077, India

**Keywords:** direct pulp capping, dentin regeneration, dentin-bridge formation, reparative dentin, calcium hydroxide, mineral trioxide aggregate

## Abstract

Background: Regenerative endodontics aims to restore normal pulp function in necrotic and infected teeth, restoring protective functions, such as innate pulp immunity, pulp repair through mineralization, and pulp sensibility. The aim of this systematic review was to assess the dentin regeneration efficacy of direct pulp capping (DPC) biomaterials. Methods: The literature published between 2005 and 2021 was searched by using PubMed, Web of Science, Science Direct, Google Scholar, and Scopus databases. Clinical controlled trials, randomized controlled trials, and animal studies investigating DPC outcomes or comparing different capping materials after pulp exposure were included in this systematic review. Three independent authors performed the searches, and information was extracted by using a structured data format. Results: A total of forty studies (21 from humans and 19 from animals) were included in this systemic review. Histological examinations showed complete/partial/incomplete dentin bridge/reparative dentin formation during the pulp healing process at different follow-up periods, using different capping materials. Conclusions: Mineral trioxide aggregate (MTA) and Biodentine can induce dentin regeneration when applied over exposed pulp. This systematic review can conclude that MTA and its variants have better efficacy in the DPC procedure for dentin regeneration.

## 1. Introduction

Vital dental pulp exposure may be caused by caries removal (caries exposure), cavity preparation where pinpoint exposure to the dental pulp (mechanical exposure), and accidental coronal pulp injury (traumatic exposure). The preservation of pulp vitality is important in all of these situations for tooth viability, nutrition, innervation, and immune defense. Dentin acts as a protective barrier that protects the tooth pulp from direct contact with potentially tissue-damaging external stimuli [1]. The formation of tertiary dentin in response to various noxious stimuli can increase the thickness of the dentin barrier [2]. The odontoblasts, which are the cells responsible for dentinogenesis, are found on the periphery of the dental pulp. These odontoblast cells could be destroyed due to severe external stimuli, such as deep dental caries. Consequently, the recruitment of progenitors and the induction of differentiation by odontoblast can take place, leading to the formation of newly generative cells, which are known as odontoblast-like cells [1].

In regenerative dentistry, DPC is a treatment procedure that utilizes the regenerative abilities of human dental pulp cells that has been described previously [3]. Pulp capping aims to facilitate the healing of injured pulp by using bioactive materials to ensure the formation of mineralized tissue or dentin bridge [4]. The use of this method may be a more conservative alternative to root-canal treatment in cases where the pulp has been exposed due to reversible injury or does not exhibit symptoms of inflammation [5]. Numerous studies were conducted to assess the effectiveness of the DPC materials with the following outcomes: pulp vitality, dentin-bridge formation, inflammation, and presence of bacteria [6,7,8]. Above all, histological analysis remains the reference standard for determining the status of the pulp and dentin-bridge formation [9]. Recently, calcium silicate–based cement has been considered as the most suitable material for pulp capping as a surface-active hard-tissue substitute because of its excellent bioactivity and biocompatibility [3]. They are widely utilized for conservative treatment, such as direct/indirect pulp capping, apexification, apexogenesis, and the repair of furcation, due to their biocompatibility, chemical bonding with tooth structure, easy handling characteristics, and good sealing ability [10].

In terms of clinical outcomes and hard-tissue formation, materials used for DPC in the exposed pulp have been evaluated [11,12,13,14]. Calcium hydroxide (CH), which has been the gold standard of care for these procedures for a long time, has antibacterial properties, and promotes healing and repair; however, it has poor sealing ability and less homogenous reparative dentin formation compared with primary dentin [1]. Several materials have been used in the past decades, such as zinc oxide eugenol, glass ionomer cement, adhesive resin, mineral trioxide aggregate, Biodentine, and enamel matrix derivatives, which have been shown to promote healing of pulp, whereas others do not have strong recommendations for use in clinical trials that indicate poor outcomes [15].

Since it was performed, many materials have been used for DPC, but what material should be ideal for DPC in dentin regeneration is still unclear. Numerous randomized and non-randomized studies with brief follow-up periods were carried out, which are insufficient to distinguish the long-term effects of different DPC-materials. By summarizing those studies, this review will recommend the most suitable materials for DPC in managing dentin regeneration, which will be useful information for dentists. The aim of this systematic review of the literature is to answer the research question: “Which biomaterial is more effective for dental capping in terms of dentin regeneration?”

## 2. Materials and Methods

### 2.1. Search Strategy

This systematic review was performed in accordance with the PRISMA (Preferred Reporting Items for Systematic Reviews and Meta-Analysis) guidelines [16]. Five electronic databases were searched for articles: PubMed, Web of Science, Science Direct, Google Scholar, and Scopus. For the search, the following keyword combinations were used: (direct pulp capping [Title/Abstract]) AND (dentin regeneration [Title/Abstract]) AND (reparative dentin [Title/Abstract]) AND (dentin bridge formation [Title/Abstract]) AND (mineral trioxide aggregate [Title/Abstract]) OR (MTA [Title/Abstract]) AND (calcium hydroxide [Title/Abstract]) AND (biodentine [Title/Abstract]). All the authors reached a consensus on the search strategy. Then three independent authors pre-selected the articles as per titles and abstracts and submitted them for the other authors’ approval. After completing this extraction, four independent and experienced authors critically checked, extracted, and confirmed the data. Articles from 2005 to 2021 were reviewed, and the literature published until 2021 was systematically searched. The search encompassed articles (full text) that have been published in peer-reviewed journals and written in English related to DPC material used for dentin regeneration.

### 2.2. Study Selection

Here, the primary concern was finding out the type of DPC material, formation/regeneration of dentin, quality of dentin formation, and outcomes. The case reports and the letters to the editors were also excluded from this review. The titles and abstracts of identified studies were independently evaluated to ensure if the studies met the inclusion criteria.

### 2.3. Inclusion and Exclusion Criteria

Inclusion criteria included the following:

1. Clinical controlled trials (CCTs), randomized controlled trials (RCTs), and animal studies;
2. Studies on permanent teeth in clinical conditions;
3. Direct pulp capping.

Exclusion criteria included the following:

1. Indirect pulp capping;
2. Total pulpotomies;
3. Deciduous/primary dentition;
4. Studies with insufficient information;
5. Non-English publications.

### 2.4. Data Extraction and Organization

Data extraction was performed on the studies that met the inclusion criteria. The following data were collected: the first author’s name; the year of publication; the age range; sample size; where this research was carried out; type of teeth; intervention/control material used for DPC; and follow-up, finding, and outcomes. The data were extracted and double-checked by the four independent authors, using a standard format. Disagreements during data extraction were resolved by means of discussion and consensus by a fifth author (MKA).

### 2.5. Quality Assessment

The Cochrane collaboration’s tool [17] for human studies and SYRCLE’s risk of bias tool [18] for animal studies were used to assess the methodological quality. For human studies, the following 6 domains were assessed: sequence generation, allocation concealment, blinding of participants and personnel, blinding of outcome assessment, incomplete outcome data, and selective reporting. For animal studies, the following 5 studies were assessed: sequence generation, baseline characteristics, blinding of outcome assessment, incomplete outcome data, and selective reporting. Using the Revman software, version 5.3, each domain was evaluated for a low, unclear, and high risk of bias.

## 3. Results

### 3.1. Selection of Studies

**Figure 1:**
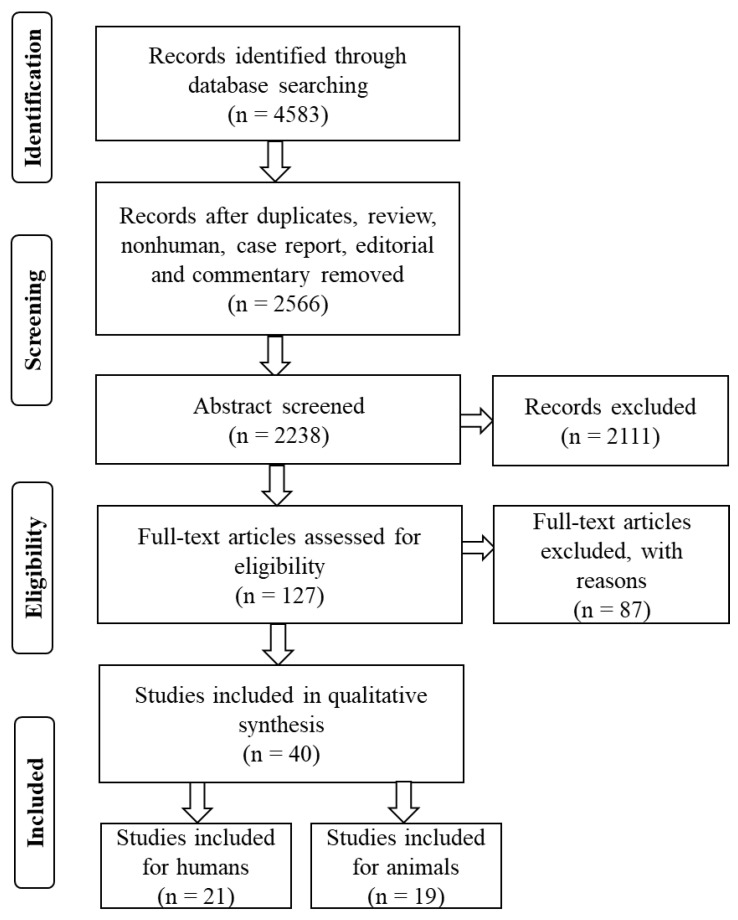
Flowchart of the systematic review.

A total of 4583 papers from databases, including PubMed, Web of Science, Science Direct, Google Scholar, and Scopus, were initially identified by using this research search strategy. After removing the 2017 articles from consideration (duplicate studies, review, case repots, editorial letters, and comments), a second round of screening was conducted on the 2566 papers that remained. A total of 127 studies were considered worthy, and 87 studies were excluded because of an unacceptable data format. Thus, 40 studies (21 humans and 19 animals) were included in this study (Figure 1), with the complete text of all of the included studies being obtained based on the research goal and inclusion and exclusion criteria.

### 3.2. Study Characteristics

Table 1 summarizes the key characteristics of the human studies that were included in this systematic review. All of the included studies were journal articles and were conducted on adults. Among these 21 studies, five are from India, four from Brazil, three from Poland, and two from Egypt. Iran, Japan, China, Korea, UK, USA, and Turkey each had one study.

Table 2 summarizes the key characteristics of the animal studies that were included in this systematic review. All of the included studies were journal articles and were conducted on animals. Among these 19 studies, five are from Japan, four from China, two Thailand, and two from Greece. Egypt, Korea, Portugal, Belgium, Tehran, and Germany had one each.

### 3.3. Risk of Bias

**Figure 2:**
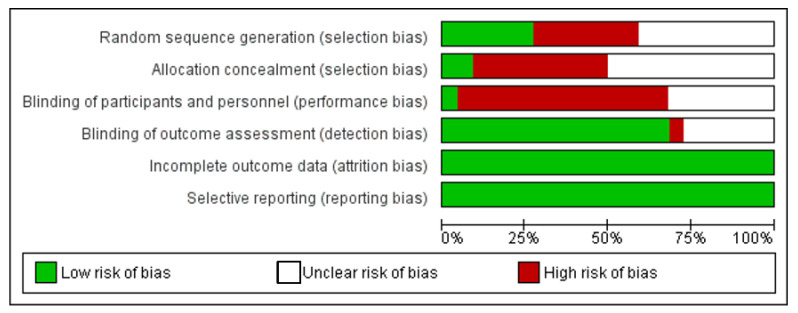
Risk-of-bias graph: review authors’ judgement about each risk of bias item presented as percentage across all included human studies.

**Figure 3:**
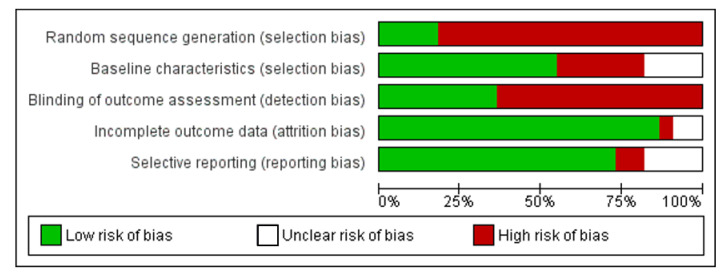
Risk-of-bias graph: review authors’ judgement about each risk of bias item presented as percentage across all included animal studies.

Figure 2 summarizes the assessment of the risk of bias in the included human studies. All assessed studies exhibited low attrition and reporting bias, whereas selection bias (random sequence and allocation concealment) and performance bias had a high and unclear risk of bias. Figure 3 summarizes the assessment of the risk of bias in the included animal studies. Studies exhibited low selection (baseline characteristics), attrition, and reporting bias, whereas selection bias (random sequence) and detection bias had a high risk of bias.

## 4. Discussion

The aim of this current systematic review was to assess the efficacy of various DPC materials that are used in dentin regeneration. This systematic review employed a risk of bias assessment of the included studies, which revealed that some of them had poor methodological quality. These studies investigated different age groups, gender, and tooth type at the population level. The quality of the included studies ranged from low to moderate, and many of them were associated with a high risk of bias. The primary goal of DPC is to maintain the pulpal tissue’s full integrity under various pathological conditions of exposure [57]. An ideal DPC material should not cause pulpal inflammation, which can lead to necrosis, and should regenerate good quality dentin at the exposure area [4]. It has been demonstrated that the use of calcium silicate–based materials as DPC agents can effectively treat dental pulp. CH has long been regarded as the gold standard of DPC material because of its biocompatibility, high pH, antibacterial effect, and ability to form a new dentin bridge at the exposure site [57]. The use of CH as a DPC material was proven to have a higher clinical success rate according to studies that followed patients for more than 10 years [58]. CH has high alkalinity, which leads to necrosis and inflammation to the pulp [9]. Besides its high solubility and lack of adhesion with hard tissues, it does not provide an optimal seal, even though the dentin bridge appears to be fully formed by the time of its complete dissolution [59,60]. CH presents tunnel defects in the dentin bridge, but there is evidence to suggest that the appearance of these defects improves with increased dentin-bridge thickness [57]. In comparison to CH, MTA has a higher rate of clinical success and can result in dentin-bridge formation that is much thicker [61,62]. Based on calcium oxide, CH and MTA both react to carbon dioxide in tissues, which is a similar mechanism of action. At the exposure site, calcite granulations are formed, and fibronectin accumulates, promoting cellular migration, proliferation, adhesion, and differentiation, resulting in the formation of hard tissue [57,63]. Bioactive molecules are released during this process that facilitate regeneration of dental pulp and are integrated into the dentin matrix during the process of dentinogenesis [64,65,66].

Nowadays, MTA has proven to be a suitable choice for DPC material because of its good sealing ability and biocompatibility [4]. Studies demonstrated that dentinal-bridge formation by MTA was higher quality, less porous, thicker, and caused less pulpal inflammation than CH [3]. Furthermore, MTA has been demonstrated to induce adhesion, migration, and attachment of undifferentiated cells in order to form a dentinal bridge while having minimal inflammatory effect on the pulp [5,67]. However, the MTA has some disadvantages, including high cost, difficulty in handling, and long setting time [4]. In a practice-based research network, confirmatory evidence for MTA’s superior performance as a DPC agent emerged when it was compared to CH in a randomized clinical trial [68]. The dental pulp capped with MTA had a 92.5 to 97.96 percent success rate in clinical trials, according to a review of the few clinical observations [69,70]. According to a histological study, MTA application directly affects the dental pulp’s regeneration potential and is associated with an increase in TGF-1 secretion by the pulp cells [71]. These cells migrate to the material–pulp interface, where they are stimulated to differentiate into odontoblastic cells, which secrete reparative dentin, affecting the quality of the dentin-bridge formation [72]. Further histological examination revealed that the hard-tissue barrier formation after DPC with MTA is not the result of the differentiation of true odontoblast and does not have the properties of regular dentin [73]. These findings recommend that the calcified tissue formation should be considered as a reparative process rather than a real regeneration process. As a result, regular dentin could not be regenerated, and a fast-setting pulp-capping material could not be used in regenerative dentistry, due to its inadequate bioactive potential [72]. Furthermore, MTA and Biodentine, in contrast to calcium hydroxide, have favorable metabolic activity and stimulate almost similar desired cellular response, resulting in a higher rate of clinical success [74]. When comparing the MTA and Biodentine groups in terms of the formation of dentin bridge, micro-CT imaging demonstrated that the MTA group had a more regular pattern of reparative dentin layer which is homogenous and uniform thickness. These findings revealed that both MTA and Biodentine have the ability to induce the dentin-bridge formation, with MTA being the most effective at improving the quality of dentin [75]. Therefore, MTA is the preferred material for DPC [72].

Biodentine is a newer calcium silicate–based DPC material having properties similar to CH and MTA, as well as favorable effects on the dental-pulp cells that promote the formation of tertiary reparative dentin [62]. By releasing TGF-β1 and stimulating odontoblasts, Biodentine promotes pulpal healing and mineralization [3,76]. Biodentine also releases silicon ions that play a significant role during the process of mineralizing the dentinal bridge [3]. It has been demonstrated that the formation of the dentin bridge by Biodentine is similar to that of MTA with no pulpal inflammatory response [77]. This is due to the anti-inflammatory effect, which inhibits the secretion of pro-inflammatory substances and reduces the recruitment of inflammatory cells [77]. According to Nowicka et al.’s findings, Biodentine and MTA induced homogeneous reparative dentin formation, whereas CH induced a more porous formation, implying that calcium silicates induce higher tissue-repair efficacy as compared to CH [27]. Jalan et al. found similar superior outcomes for dental pulp capped with Biodentine when compared to Dycal [26]. Therefore, Biodentine material has great potential as a pulp-capping agent because of its proper setting time and restorative properties. However, studies suggested that long-term clinical research is still required to check the efficacy of Biodentine [3].

Adhesive systems have been investigated as suitable DPC materials because of their ability to adhere to dentin to protect the pulp from bacterial contamination [1]. On the other hand, bonding agents have been shown to have direct cytotoxic effects on dental-pulp cells [1]. These materials did not show favorable responses when compared to MTA in terms of pulpal inflammation and hard-tissue formation [57]. A new dentin-bridge formation was observed in all MTA specimens, whereas no hard-tissue deposition was observed even if the pulp tissue showed no symptoms of inflammation in the polymeric-based materials group, or the adhesive materials group only induced a few hard-tissue depositions with pulpal necrosis and inflammation [78,79].

## 5. Conclusions

The findings of this systematic review, based on the available information, conclude that MTA and its variants have a higher success rate in dentin regeneration. MTA and its variants are more likely to form a homogenous dentinal bridge than CH and other DPC materials.

## Figures and Tables

**Table 1:** Characteristic of the studies included (humans) in the systematic review.

Author (Year)	Country	Type of Study	Age	Type of Teeth	Experimental Materials	Comparing Materials	Follow-up	Outcomes
Cobanoglu et al. 2021 [19]	Turkey	Controlled clinical trial	23–35	Third molars	Clearfil Protect Bond	Clearfil SE Bond and CH	90 days	CH group showed better hard-tissue formation than the experimental group.
Sharma et al. 2021 [20]	India	Controlled clinical trial	15–30	Premolars	Endosequence Root Repair Material and Endocem MTA	ProRoot MTA	30 days	The mean thickness of dentin-bridge formation in ProRoot MTA was greater than the other two experimental groups.
Holiel et al. 2021 [21]	Egypt	Controlled clinical trial	15–25	Premolars	Treated dentin matrix hydrogel	Biodentine and MTA	2 weeks and 2 months	Complete dentin-bridge formation was observed with numerous dentinal tubule lines showing a positive trend to dentin regeneration.
Holiel et al. 2021 [22]	Egypt	Randomized clinical trial	18–40	Permanent posterior teeth	Treated dentin matrix hydrogel	Biodentine and MTA	3, 6, 12, 18, and 24 months	Dentin-bridge formation was significantly superior of a higher thickness than Biodentine and MTA.
Hoseinifar et al. 2020 [23]	Iran	Randomized clinical trial	14–25	Premolars	Calcium-enriched mixture	MTA and Biodentine	6 weeks	No significant differences were observed between the groups in terms of the dentine bridge formation.
Suzuki et al. 2019 [24]	Japan	Controlled clinical trial	18–33	Third molars	CO_2_ laser irradiation	Dycal	6 and 12 months	Self-etching adhesive system following CO_2_ laser irradiation without carbonization of the exposed pulp demonstrated dentin-bridge formation that was comparable to Dycal.
Mahendran et al. 2019 [25]	India	Controlled clinical trial	18–24	Premolars	Simvastatin + α-TCP and atorvastatin + α-TCP	MTA	7, 30, and 90 days	No significant difference was observed in terms of hard-tissue formation between the groups.
Jalan et al. 2017 [26]	India	Randomized clinical trial	15–25	Premolars	Biodentine	CH	45 days	Dentin-bridge formation was significantly thicker and more continuous with Biodentine in comparison to Dycal.
Nowicka et al. 2016 [11]	Poland	Controlled clinical trial	19–30	Third molars	Single-bond universal	CH	6 weeks	Single-bond universal showed less dentin-bridge formation than CH.
Nowicka et al. 2015 [27]	Poland	Controlled clinical trial	19–32	Third molars	MTA, Biodentine, single-bond universal	CH	6 weeks	MTA and Biodentine groups showed significantly higher dentin-bridge formation than CH and single-bond universal groups.
Swarup et al. 2014 [28]	India	Controlled clinical trial	11–15	Premolars	Nano hydroxyapatite	MTA, CH	15 and 30 days	Continuous dentin-bridge formation was observed in the nano hydroxyapatite and MTA groups. Only MTA group showed regular pattern of dentinal tubules.
Parolia et al. 2010 [29]	India	Controlled clinical trial	15–25	Premolars	Propolis, MTA	Dycal	15 and 45 days	Propolis and MTA showed more dentin-bridge formation than Dycal group.
Accorinte et al. 2008 [30]	Brazil	Controlled clinical trial	15–30	Premolars	Clearfil LB 2V and Clearfil SE Bond	CH	30 and 90 days	Few specimens showed dentin-bridge formation in the experimental group, whereas CH showed dentin-bridge formation almost all the specimens.
Accorinte et al. 2008 [31]	Brazil	Controlled clinical trial	15–30	Premolars	MTA	CH	30 and 60 days	CH showed faster hard-tissue formation compared to MTA and a similar response with the hard-tissue bridge in almost all cases was observed.
Accorinte et al. 2008 [32]	Brazil	Controlled clinical trial	15–30	Premolars	MTA	CH	30 and 60 days	Dentin-bridge formation was lower in the CH group compared to MTA group.
Sawicki et al. 2008 [33]	Poland	Controlled clinical trial	10–18	Immature premolars	WMTA	CH	47–609 days	Complete, thicker, and more solid dentin bridge was observed in the WMTA group when compared with CH.
Lu et al. 2008 [34]	China	Controlled clinical trial	20–25	Third molars	Clearfil SE Bond	CH	7, 30, and 90 days	The dentin-bridge formation in the experimental group was significantly lower compared to CH group.
Min et al. 2008 [35]	Korea	Controlled clinical trial	21–50	Third molars	MTA	CH	2 months	The thickness of the dentin-bridge formation in the MTA group was statistically greater than CH group.
Nair et al. 2006 [36]	UK	Randomized controlled trial	18–30	Third molars	MTA	Dycal	1 week, 1 month, and 3 months	Complete hard-tissue formation was observed in the MTA group, whereas less consistent formation of hard-tissue barrier with numerous tunnel defect was observed in the Dycal group.
Silva et al. 2006 [37]	Brazil	Controlled clinical trial	12–20	First premolars	Single-bond adhesive system	CH	30 days	No dentin formation at the exposure area in the single-bong adhesive system group, whereas dentin-bridge formation was observed in the CH group.
Iwamoto et al. 2006 [38]	USA	Controlled clinical trial	18–60	Third molars	WMTA	CH	136 ± 24 days	WMTA showed a dentin-bridge formation similar to CH’s.

CH, calcium hydroxide; MTA, mineral trioxide aggregate; WMTA, white mineral trioxide aggregate; α-TCP, α-tricalcium phosphate.

**Table 2:** Characteristic of the studies included (animals) in the systematic review.

Author (Year)	Country	Animal	Age/ Weight	Type of Teeth	Experimental Materials	Comparing Materials	Follow-up	Outcomes
Islam et al. 2021 [4]	Japan	Wister rats	8–9 weeks	Maxillary first molar	Phosphorylated pullulan + MTA	MTA, Super Bond	1, 3, 7, and 28 days	The experimental group showed more homogenous mineralized tissue formation compared to MTA and Super bond groups.
Yoon et al. 2021 [39]	Korea	Sprague-Dawley rats	6–8 weeks	Maxillary first molar	Osteostatin + ProRoot MTA	ProRoot MTA	4 weeks	The combined material group showed more mineralized dentin-bridge formation compared to ProRoot MTA group.
Trongkij et al. 2019 [40]	Thailand	Wister rats	8 weeks	Maxillary first molar	Bio-MA	WMTA	1, 3, and 30 days	Complete dentin-bridge formation was observed in the Bio-MA group which is similar to WMTA.
Hanada et al. 2019 [41]	Japan	Wister rats	9 weeks	Maxillary first molar	Bioactive glass	Dycal and WMTA	1, 4, and 7 days	Bioactive-glass-based cement induced a significant level of reparative dentin formation, similar to MTA.
Takahashi et al. 2019 [42]	Japan	Wister rats	9 weeks	Maxillary first molars	S-PRG filler	MTA	1, 2, and 4 weeks	S-PRG filler showed to promote tertiary dentinogenesis, which is similar to MTA.
Paula et al. 2019 [43]	Portugal	Wister rats	12–14 weeks	First mandibular molars	WMTA and Biodentine	Positive control (exposure without treatment)	3, 7, and 21 days	Mineralized tissue formation was observed in the WMTA and Biodentine group. Biodentine may lead to the formation of pulp calcifications.
Li et al. 2018 [44]	Belgium	Minipigs	33–35 months	Incisors, canines, premolars and molars	Tricalcium silicate cement	ProRoot MTA and TheraCal	70 days	Complete reparative dentin formation with tubular structures was observed in the tricalcium silicate and ProRoot MTA groups.
Trongkij et al. 2018 [45]	Thailand	Wister rats	8 weeks	Maxillary first molar	Bio-MA	WMTA	1 and 7 days	Bio-MA can stimulate reparative dentin formation which is similar to WMTA.
Shinkai et al. 2017 [46]	Japan	Sprague-Dawley rats	8–9 weeks	Maxillary first molar	All-in-one adhesives (Clearfil Tri-SBond ND, G Bond Plus, Bond Force, Adper Easy Bond, Xeno V)	MTA	14 days	MTA group showed complete dentin-bridge formation, whereas all-in-one adhesives group showed incomplete or partial dentin-bridge formation.
Negm et al. 2017 [47]	Egypt	Dogs	4–6 months	Four teeth in three quadrants	Portland cement + 10% calcium hydroxide + 20% bismuth oxide, Portland cement + bismuth oxide	MTA	3 weeks and 3 months	Addition of calcium hydroxide to Portland cement improves the dentin-bridge formation qualitatively and quantitatively.
Shi et al. 2016 [48]	China	Beagle dogs	8 months	Maxillary and mandibular incisors	iRoot BP Plus	MTA	3 months	Both experimental groups showed complete calcified bridge formation with no significant difference.
Suzuki et al. 2016 [49]	Japan	Sprague-Dawley rats	6 weeks	Maxillary first molar	Adhesive resin – Primer I, II and III	Dycal	14, 28, 56, and 112 days	Higher quality of the mineralized tissue formation was observed in the experimental groups.
Liu et al. 2015 [50]	China	Wister rats	180–200 g	Maxillary first molars	iRoot BP Plus	MTA	1 and 4 weeks	iRoot BP Plus induced the formation of reparative dentin bridge.
Tziafa et al. 2014 [51]	Greece	Miniature swine	18 months	Incisors, canines, premolars and molars	Biodentine	MTA angelus	3 and 8 weeks	The thickness of hard-tissue bridge formation was significantly higher in the Biodentine group.
Danesh et al. 2012 [52]	Tehran	Dogs	18–24 months	Canine	Biomimetic carbonated apatite	MTA	7 and 70 days	Biomimetic carbonated apatite did not induce hard-tissue bridge formation.
Dammaschke et al. 2010 [53]	Germany	Wister rats	3 months	Maxillary first molars	Reculcin AquaPrime+ monoBond, ScotchBond 1, Gluma Comfort Bond	CH	1, 3, 7, and 70 days	CH showed more frequent reparative dentin formation than the experimental groups.
Cui et al. 2009 [54]	China	Dog	1.5 years	Incisor, canine, premolars and first molar	Clearfil SE Bond, Imperva FluoroBond, Prompt L-Pop	Dycal	7, 14, and 30 days	Hard-tissue formation was observed in the experimental group.
Lu et al. 2006 [55]	China	Beagles	1 year	All teeth	Clearfil SE Bond	CH	7, 30, and 90 days	Dentin-bridge formation was less in the experimental group than CH.
Koliniotou-Koumpia and Tziafas 2005 [56]	Greece	Dog	2.5–3.5 years	Maxillary and mandibulary molars, premolars, canines, and third incisors	Clearfil SE bond, Prompt L-pop, Etch and prime 3.0, single-bond	Dycal	7, 21, and 65 days	Continuous hard-tissue bridge formation was totally absence in the experimental groups.

CH, calcium hydroxide; MTA, mineral trioxide aggregate; WMTA, white mineral trioxide aggregate; CaCl2, calcium chloride.

## Data Availability

The authors would like to exclude the data availability statement.
